# Current concepts of Harm–Benefit Analysis of Animal Experiments – Report from the AALAS–FELASA Working Group on Harm–Benefit Analysis – Part 1

**DOI:** 10.1177/0023677216642398

**Published:** 2016-05-17

**Authors:** Aurora Brønstad, Christian E Newcomer, Thierry Decelle, Jeffrey I Everitt, Javier Guillen, Kathy Laber

**Affiliations:** 1 University of Bergen, Department of Clinical Medicine, Bergen, Norway; 2 AAALAC International, Frederick, MD, USA; 3 Chief Veterinary Officer, Sanofi, Marcy l’Etoile, France; 4 Department of Laboratory Animal Science, GlaxoSmithKline, Research Triangle Park, NC, USA; 5 AAALAC International, Pamplona, Spain; 6 Chief, Comparative Medicine Branch, NIEHS/NIH, Research Triangle Park, NC, USA

**Keywords:** harm–benefit, ethical review, animal experiment

## Abstract

International regulations and guidelines strongly suggest that the use of animal models in scientific research should be initiated only after the authority responsible for the review of animal studies has concluded a well-thought-out harm–benefit analysis (HBA) and deemed the project to be appropriate. Although the process for conducting HBAs may not be new, the relevant factors and algorithms used in conducting them during the review process are deemed to be poorly defined or lacking by committees in many institutions. This paper presents the current concept of HBAs based on a literature review. References on cost or risk benefit from clinical trials and other industries are also included. Several approaches to HBA have been discovered including algorithms, graphic presentations and generic processes. The aim of this study is to better aid and harmonize understanding of the concepts of ‘harm’, ‘benefit’ and ‘harm–benefit analysis’.

## Introduction

The growing body of international agreements, regulations and guidelines pertaining to the use of animals in research emphasizes that such use is a privilege granted by society to the research community to facilitate scientific advancement under the condition that animal use is necessary, and conforms to principled and effective animal welfare procedures.^1–4^ Use of animals in research is generally accepted by policy makers through regulations, and is based on the presumption that harm–benefit analysis (hereafter HBA) warrants such use. The framework of acceptance has been described in the regulations of the EU Directive 2010/63^2^ and in the guidelines described in the *US National Research Council Guide for the Care and Use of Laboratory Animals*, 8th edition (hereafter *NRC Guide*).^1^ Typical requirements that must be met when using animals include application of the 3Rs (see Box 1),^5^ and some judgment of the likelihood that the outcome of each project will contribute to the core scientific information that ultimately produces benefits and offers the prospect of sustaining or enhancing human and animal lives, as well as protecting the earth’s ecosystems.^2^

**Box 1.** The 3Rs of Russell and Burch.

**Replacement**: Substitution for conscious living higher animals of insentient animals, or methods not involving animals.
**Reduction**: Reduction in the numbers of animals used to obtain information of a given amount and precision.
**Refinement**: Decrease in the incidence or severity of inhumane procedures.
Source: Russell WMS and Burch RL. *The principles of humane experimental technique*. London: Methuen, 1959.

The need to perform an HBA has been explicitly mentioned in EU Directive 2010/63,^2^ the Office International des Epizooties (OIE) *Terrestrial Animal Code*^4^ and the Council for International Organizations of Medical Sciences–International Council for Laboratory Animal Science (CIOMS–ICLAS) *International Guiding Principles for Biomedical Research Involving Animals*,^3^ and is implied in the *NRC Guide*.^1^ The Association for Assessment and AAALAC International has communicated its expectation to all programs participating in accreditation that an HBA based upon the EU Directive should be performed at least in some instances, and underlined advice in the *NRC Guide* indicating that ‘the IACUC is obliged to weigh the benefits of the study against potential animal welfare concerns’.^1^ More widely, 178 countries participated in the World Assembly of Delegates to the World Organization for Animal Health (OIE) which recently revised the *Terrestrial Animal Health Code* to include Chapter 7.8 (Use of animals in research and education) under Section 7 (Animal welfare).^4^ Chapter 7.8 emphasizes the importance of HBA and anticipates inclusion of HBA in the national guidelines of signatory countries by agreement, although there is no legal mandate that would compel them to do so. The European Science Foundation explicitly states that the use of animals must be based on an HBA in a policy document.^6^ Also, the *International Guiding Principles for Biomedical Research Involving Animals* have recently been revised in a partnership between CIOMS (which is sponsored by the World Health Organization and UNESCO as well as 170 other international scientific organizations) and the ICLAS (which also enjoys broad international representation).^3^ Although this document is also not legally-binding, it serves as a further widely used influential reference reinforcing a globally unified front on the importance of the HBA approach in scientific research using animals.^7^

Both the USA and Europe have designated responsible entities (institutional/regional/national animal ethics committees (AECs) or institutional animal care and use committees (IACUCs)) and charged them with responsibility for project evaluation including HBA.

HBA is based on an ethical stance that expects each scientific endeavor involving research animals to be planned and executed so that harms to animals are minimized and potential benefits from animals are maximized.^8^

The use of animals in research raises ethical questions on the subject of harm, the research objectives and the targeted recipients of the benefit. Animal experiments provide essential knowledge that cannot be achieved by alternative methods, and consequently using some animals to maximize utility for humans, other animals or the environment is inevitable. However, the fact that animal experiments are often useful and essential does not justify the use of animals without ethical qualification in all cases. Historical success of animal experimentation is not sufficient to justify continued animal use, as science is constantly evolving and alternative methods can become available. A justification cannot be reasonably applied as a universal law or as a tool for sweeping and categorical acceptance; as some evaluation of a particular case is expected by the scientific community and by the public where there is a potential for animal harm. Case-by-case evaluation is common practice^9^ and not only because it is anchored in regulations and guidelines.^1,2^ People apply harm–benefit information in decision-making whether or not they are in favor of a particular type of animal experiment and this applies whether they have positive or negative attitudes to animal experiments.^10^ However people still vary in their understanding of HBA principles and will therefore likely apply them differently.

The aim of the American Association for Laboratory Animal Science–Federation of European Laboratory Animal Science Associations (AALAS–FELASA) working group (WG) on harm–benefit analysis is to promote common understanding of the principles and approaches to HBA as an important element in the ethical evaluation of the use of animals in the USA^1^ and Europe.^11^ Such common understanding and practices might also build confidence in data exchange and collaboration on animal research.

Terms of reference were defined for the AALAS–FELASA WG on HBA (see Box 2). This paper presents the results of tasks 1 and 2, i.e. to review the existing literature and define and describe the current concepts and elements of HBA.

**Box 2.** Terms of reference of the AALAS–FELASA working group on harm–benefit analysis.

1.	Review the existing literature on harm–benefit analysis.
2.	Define and describe the current concepts and elements of harm–benefit analysis.
3.	Recommend how it can be addressed by persons responsible for the protocol/project applications.
4.	Define how harm–benefit analysis can be implemented by responsible entities as part of the ethical evaluation.
5.	Present practical cases that may exemplify common situations in the research environment.

This presentation is based upon the main findings in the literature reviewed of relevance to HBA and the approaches used to systemize HBA and reflect the current understanding of harm, benefit and HBA.

## Methods – review of existing literature on HBA

The literature search included publications on harm–benefit or cost–benefit evaluations of the use of animals in research, education and testing. We also included some material on cost/harm/risk–benefit analysis from human medical trials^12^ as well as studies on risk–benefit perceptions in general.^13–15^ Guidelines and policy statements on the use of animals in research and education (by for example CIOMS, ICLAS, OIE, US Government, European Commission, FELASA and AALAS) were also reviewed.

## Results – findings

In the following section we describe how harm and benefit have been characterized and summarize the methods used to compare and weigh harms and benefits.

The cost–benefit evaluation was discussed by Bateson in 1986 in connection with animal research when he introduced the ‘Bateson cube’ as a model to illustrate the concept.^16^

The term ‘cost’ has been rejected by some authors as it evokes negative associations with economic cost, and ‘cost’ has therefore been replaced by ‘harm’ to make it clearer that it is the negative impact for the animals that is relevant in the ethical evaluation of animal experiments.^7,23^ Furthermore, in economic discussions, ‘cost’ and ‘benefit’ can be measured in a common currency which has no parallel in research animal studies. In animal studies the subjects potentially experience harm measured in the currency of pain and distress, and the potential benefits, which are often difficult to measure, redound to a different set of individuals (or species) in another category. Comparing apples and oranges is another metaphor used recently to illustrate the difficulty of comparing these two different concepts.^24^

The use of the term ‘risk–benefit’ appears to be disappearing in the ethical review of animal experiments, compared with references to ‘harm–benefit’ in human studies. The willingness to take a risk seemingly acknowledges the patient’s recognition of a potentially accrued benefit, thus diminishing the contrast of the coupling, ‘risk–benefit’. We found the ideas from several human medical trials^12,25,26^ to be very interesting and worthy of inclusion in our discussion of HBA in animal studies. This also applies for risk–benefit evaluations in other fields.^13–15,27^ In the end there will be people evaluating information and making decisions.

## Harm

The WG reviewed the available literature on HBA in animals and key documents on the subject of harm. Different domains or factors that may impair animals and which are relevant to the consideration of harm were identified. Literature suitable to the construction of a helpful framework for a systematic HBA were also selected. The different ‘harm’ factors that were identified are summarized in Table 2. The harm factors can be subgrouped as ‘animal welfare harms’, ‘animal rights^28^ harms/intrinsic nature harms’ and ‘quality harms’, where ‘animal welfare harms’ is the largest subgroup as shown in Table 2.

**Table 1.** Similarities and differences for project evaluation and authorization in the USA and EU.

	US USDA Animal Welfare Act	US PHS Policy and the Guide for the Care and Use of Laboratory Animals	EU Directive
Responsible body for protocol/project evaluation	Institutional Animal Care and Use Committee (IACUC)	Institutional Animal Care and Use Committee (IACUC)	Public Competent Authority As this function can be delegated to other bodies: a number of Member States allow local animal welfare bodies/ethics committees and/or external ethics committees to perform the evaluation
Minimum composition evaluation body	Three members including a DVM and a non-affiliated member	Five members including a DVM, a practising scientist, an expert in non-biological science and a non-affiliated member	Expertise in: (a) the areas of scientific use for which animals will be used including replacement, reduction and refinement in the respective areas; (b) experimental design, including statistics where appropriate; (c) veterinary practice in laboratory animal science or wildlife veterinary practice where appropriate; (d) animal husbandry and care, in relation to the species that are intended to be used
Harm/benefit analysis	–	IACUC is obliged to weigh the objectives of the study against potential animal welfare concerns	A harm–benefit analysis of the project, to assess whether the harm to the animals in terms of suffering, pain and distress is justified by the expected outcome taking into account ethical considerations, and may ultimately benefit human beings, animals or the environment
Responsible body for protocol/project authorization	IACUC	IACUC	Public Competent Authority. Only Belgium allows institutional ethics committees to approve projects that they have evaluated favorably
Other bodies involved	–	–	Local (institutional) animal welfare body (AWB). This includes the person(s) responsible for the welfare and care of animals, a scientist (in the case of users) and input from the designated veterinarian
Post-approval monitoring	Yes, by IACUC	Yes, by IACUC	Yes, AWB to follow the development and outcome of projects
Retrospective assessment	–	–	Yes, at least for all projects using non-human primates and projects involving procedures classified as ‘severe’. Assigned to the competent authorities, but it can be delegated to

**Table 2.** Harm factors identified in literature review.

							Animal welfare harms												Animal rights harm		Quality harms	
Ref.	Species, choice } \rot \hbox{of animals	Sentience and consciousness	Quality of animals	Duration	Duration related to lifespan	Number of animals	Origin, acquisition }\cr \rot \hbox{or transport	Care, housing factors, }\cr \rot \hbox{handling, health care	Possibility to express }\cr \rot \hbox{normal behavior	Staff competence and quality	Hunger and thirst	Discomfort	Pain, injury or disease	Fear, anxiety and distress	Frequency of procedures	Severity	Risk of harm}\cr \rot \hbox{=\,probability}\cr \rot \hbox{\times severity	Deaths (caused by }\cr \rot \hbox{the experiment)	Intrinsic value and }\cr \rot \hbox{animal rights	Genetic modulation }\cr \rot \hbox{- respect for nature	Aim, realistic potential }\cr \rot \hbox{scientific quality	Non-publishing }\cr \rot \hbox{of negative results
Bateson 1986^1,6^								+						+							+	
Bateson 1992^3,7^	+												+	+					+			
Porter 1992^32^	+			+	+	+		+					+								+	
Mellor 1994^30^		+							+		+	+	+	+								
Stafleu 1999^40^	+		+	+		+				+	+		+	+	+				+		+	
Mellor 2004^31^									+		+	+	+	+								
Rickard 2004^5,6^	+							+		+												
Voipio 2004^7^	+			+		+	+	+	+	+			+	+	+	+		+				+
Schuppli 2004^3,8^	+	+				+		+	+	+			+	+					+	+		
FELASA 2005^41^						+	+	+		+			+	+		+						
Degrazia 2007^4,9^							+	+			+		+	+				+				
EU Dir. 2010/63^2^	+					+	+	+					+	+		+		+				
Boddy 2011^2,6^														+			+					
Lindl 2012^60^													+	+								

Harm caused by painful procedures has been a main concern among antivivisectionists and is one major concern in public opinions of animal experiments.^10^ Pain and other impacts of nociceptive physiological processes can also have detrimental consequences for research and the validity of data. However, clinical advances increasingly allow pain and nociceptive responses to be well controlled by the use of appropriate anesthetics and analgesics. Although relevant and important, pain is not the only potential source of harm. Other factors can impact animal well-being negatively by inducing suffering and distress, warranting inclusion in the HBA discussion.

The five freedoms^29^ (see Box 4) encompass the impacts on animals in a broader perspective that are more aligned with modern regulations, guidelines and the layperson perspective than only the consideration of pain.

**Box 3.** Background and Impetus for harm benefit analysis.

The idea that HBA should precede experimentation on live subjects was originally developed in relation to experimentation on human subjects. The importance of considering the harms and benefits in relation to experimentation was first introduced in the 10 principles of the Nuremberg Code^17^ developed following the Nuremberg trials on human experimentation after World War II. The Nuremberg Code emphasizes that experiments should be such as to yield fruitful results for the good of society, unprocurable by other methods or means of study, and not random and unnecessary in nature. The Nuremberg Code also provided that experiments should be so conducted as to avoid all unnecessary physical and mental suffering and injury and experiments should assure that the degree of risk to be taken should never exceed that determined by the humanitarian importance of the problem to be solved by the experiment. Notably, the Nuremberg Code identified animal studies as a crucial first step in the protection of humans from the primary exposure to risks by establishing a results-based scientific foundation for the belief that similar anticipated results in humans will justify the performance of the experiment. Subsequent to the Nuremberg Code in the late1970s, the Belmont Report^18^ in the United States and the World Medical Association Declaration of Helsinki^19^ elaborated ethical principles for the conduct of human studies sharpening the concept that the harms and risks to human subjects participating in experiments should be evaluated in relation to the benefits accrued from participation. Quoting Principle 18 of the Declaration of Helsinki, ‘Every medical research study involving human subjects must be preceded by careful assessment of predictable risks and burdens to the individuals and communities involved in the research in comparison with foreseeable benefits to them’^19^ – interestingly similar principles were not then specified for experiments in animals.
There is a longstanding precedent for the use of animals in research for the pursuit of outcomes beneficial for humans or animals. In 1985 international principles were developed by CIOMS offering guidance to governments and scientists in countries with a broad spectrum of regulatory oversight mechanisms.^20^ Concomitantly very similar principles were adopted by the US Government,^21^ and in Europe in the preamble of the European Treaty Series (ETS) 123.^22^ In both cases the principles embraced the notion that studies in living animals should be designed and performed with due consideration of their relevance to human or animal health and the advancement of knowledge while the US guidelines also added animal studies for ‘the good of society’ as a motivation with merit.^21^ Both sources suggested that where one or more of the guiding principles potentially would be compromised by the nature of the animal study, an appropriate responsible entity or Animal Ethics Committee (AEC), rather than the investigator directly concerned with the studies, should have decision authority and that exceptions to the guidelines should not be made for teaching or demonstration purposes.^21,22^ The CIOMS statement and the US Guiding Principles statement on this are almost identical and are already summarized in the text provided. In 1985 CIOMS stated ‘VIII. Where waivers are required in relation to the provisions of article VII, the decisions should not rest solely with the investigators directly concerned but should be made, with due regard to the provisions of articles IV, V, and VI, by a suitably constituted review body. Such waivers should not be made solely for the purposes of teaching or demonstration’^20^
These guidelines served as the impetus for the later development of the HBA concept by institutional/regional/national AECs or IACUCs. However, while broadly inferring the importance of benefits, the 1985 guidelines did not specifically elaborate on the scope and kinds of benefits that might be deemed relevant. In recent years, regulatory guidelines as well as an interested public have sought greater clarity and precision in the disclosure/discussion of the benefits perceived from animal research, and public support and financial support for science would be well served by improving our efforts in this area. The guidelines were not instructive in 1985 about what constitutes a benefit and there was no additional clarification in the guideline revisions in recent years. The concept of a benefit seems to be quite broad and defining it is left to the bodies overseeing the research. The key change in the old versus the new guidelines is that the early guidelines indicated that harm should be reduced and science should be conducted for a relevant and productive purpose. Later guidelines suggested (or imposed) that committees should link and measure the harm–benefit relationship.
The United States and Member States of the EU that support research animal use have defined systems or bodies (e.g. AECs, IACUCs and other responsible entities such as national committees) that are charged with the responsibility for ensuring that animal use is in accordance with regulations and ethical norms and authorizing projects based on specific information in applications provided by the scientist conducting the research animal studies. A summary of similarities and differences for project evaluation and authorization in the USA and EU is presented in Table 1. Regulatory compliance is not just important to the responsible entities overseeing research. Compliance with regulations and operating within a consistent ethical framework are important priorities for scientists who are constantly pursing the simplest experimental model systems that yield rigorous and reliable data. Operating without a consistent ethical framework may damage the reputation of research and increases the risk of negative public perceptions of the specific research activities and the broader research enterprise.

**Box 4.** The five freedoms.

These are currently expressed as:
**Freedom from hunger or thirst:** by ready access to fresh water and a diet to maintain full health and vigor.
**Freedom from discomfort:** by providing an appropriate environment including shelter and a comfortable resting area.
**Freedom from pain, injury or disease:** by prevention or rapid diagnosis and treatment.
**Freedom to express (most) normal behavior:** by providing sufficient space, proper facilities and the company of the animal’s own kind.
**Freedom from fear and distress:** by ensuring conditions and treatment which avoid mental suffering.

These include not only harm caused by pain, but also any aspect that can compromise animal well-being, including the opportunity to express normal behavior. The five freedoms were originally defined for farm animals,^29^ and Mellor and Reid can be credited with adapting the five freedoms to discriminate between harm levels in research animals.^30,31^

Injuries and diseases are inherent harm factors relevant to many animal experiments. The negative impact of these can often be minimized or controlled by different refinements that mitigate the negative effect on animals such as the use of analgesics in the case of pain. Experimental conditions can also cause fear, anxiety and distress for animals and are also legitimate harm factors. Minor procedures alone might not cause a significant negative impact; however if they are repeated frequently or the procedure lasts for a longer period or conducted over a substantial part of the animal’s lifespan, then the total burden must be regarded as harmful for the animal. Frequent transport, single housing of social animals and impeding an animal’s ability to express normal behavior are examples of such harm factors. Transport of animals over shorter distances is not regarded as a major impact per se, but the stress of frequent transport requiring the animal to continually adapt to new locations would warrant consideration. Animals need time to rest and express normal behavior, and this can be disturbed by the imposition of frequent procedures.

Individual procedures causing a moderate negative impact can produce a severe negative influence on animals if they are repeated over a long period or if many are compressed into a short time interval.

The duration of such an impact as a proportion of the lifespan of the animal is another relevant domain in this discussion.^32^ Cumulative harm reflects the total negative impact of an animal’s experience through its whole lifespan.^33^ Harm has also been defined as a product of the probability and the severity of harm.^26^

Animals are affected by housing and the care provided. Housing facilities must be suitable to cover the animals’ needs, and the facility physical plant must be properly managed to avoid animal injuries. Simple things such as daily handling and care can be experienced as harmful if the staff members responsible are not appropriately qualified. For example, handling of fish often includes taking them out of water, their normal habitat. Being out of water is a life-threatening condition causing severe stress for the fish. The quality of care will depend very much on the competence of the staff in the animal facility, which includes their knowledge, experience, skills and motivation. Their ability to recognize any sign of negative impact on an animal and their ability to take corrective steps will make a significant difference for the animal’s experience in an experiment. The concept of care exceeds mere harm avoidance; good animal care also includes proactive actions that optimize animal well-being, as reduction of well-being is a form of harm.

All harm factors mentioned so far are dependent on sentience and the degree to which the animal is aware of its situation. Some animals are regarded as more sentient than others, making the species of animal used a relevant factor in the evaluation of harm. According to the 3R principles, replacement can be ‘substitution for conscious living higher animals of insentient animals’.^5^ The definition of what constitutes ‘conscious living higher animals’ is evolving and controversial, inviting comparisons to Singer’s discussion of speciesism.^34^ Our understanding of the conscious species with the capacity to suffer from harm depends very much on our knowledge of that species. Non-human primates are regarded as ‘conscious living higher animals’ that need extra attention by some groups. Article 8 in the EU Directive explicitly mentions the use of non-human primates reflecting a stance that doing experiments using non-human primates is regarded as more harmful than using other species. Such a stance can be discussed as some species are more easily habituated to human contact and experimental conditions than others.

Accelerated genetic predisposition to disease and putting an animal at risk of harm by developing a chronic debilitating or devastating disease may cause much distress for the animal, even if pain is not the main issue in the earlier phases of phenotype progression. This applies for some of the genetically-modified animal models that are used.

The number of animals has also been included as a dimension of harm. However, the reduction principle, or limiting the harm to a few, can sometimes be at odds with a refinement effort which results in little harm but involves a greater number of animals.^35,36^ Experimental approaches involving cumulative mild harm to many animals in lieu of more severe harm to a few animals create ethical dilemmas that warrant thoughtful analysis and resolution.^35^ Failure to respect intrinsic values has also been suggested by some authors as a harm factor.^37–40^

Professional societies have addressed the importance of recognizing harm and pursuing harm reduction in their publications. FELASA working groups^11,41^ have extensively documented different experimental procedures and offered an assessment of how these might influence animal well-being. Two AALAS Position Papers^42,43^ have also addressed harm and harm reduction in relation to the matter of judicious animal use. But none of these sources offers guidance on calculation of the HBA.

Factors related to the research animal study aim, potential for success, design of experiments and (lack of) publication of results have also been mentioned as harm factors.^16,32,40^ Animals are not harmed more by low-quality design than by high-quality design per se. Performing poor-quality experiments that are not likely to yield valuable information is an irresponsible use of animals – independently of whether the animals experience much harm or not. Poor-quality studies can cause harm if they produce misleading results.^44^ In the Bateson cube model, quality of design is presented as a separate domain or dimension – independent of harm and benefit, emphasizing the importance of this factor.^16^

### Severity classifications and discrimination of harm levels – the need for categories

From the discussion above, it can be difficult to compare circumstances across different harm domains, e.g. comparing minor surgery under anesthesia with extended housing in isolation for a social species. Different ways to evaluate overall harm, e.g. categorizing harm into severity classes,^33,45^ have been developed. The baseline of zero (0) harm is vaguely described and can be approximately equated with any procedure comparable with needle injection in Europe.^46^ Some authors define categories for each harm domain and on this basis make a cumulative/overall severity/harm score for the experiment.^30–32^ Others describe the different categories using examples of procedures.^47^ Killing animals for their organs and tissues is not considered a procedure, and thus is not subject to HBA according to European regulations.^2^ Anesthetizing an animal without any prior intervention and then euthanizing the animal under anesthesia is classified as a separate harm category under European regulations,^2^ although this requires the consideration of the applicability of alternatives (i.e. the 3Rs) for animal species covered by the US Animal Welfare Act regulations. The reason for categorizing this in a separate severity class is that the opportunity for potential negative experiences for the research animal is minimized and is effectively limited to ineffectual anesthesia,^46^ even if death is the outcome. This is controversial, and not everyone agrees that this is less severe, even if suffering is not involved,^37^ because animals have an intrinsic value.^40^

Maximum severity encompasses conditions that cannot be treated, relieved or involve death as the outcome. Such experiments should always be carefully considered regarding refinement and use of more humane endpoints.

EU Directive 2010/63 demands the classification of experiments according to the level of harm in research animal studies in order to ensure that science is public-accountable.^2^ A European Commission expert working group has identified four categories, i.e. terminal, mild, moderate and severe,^33,46^ providing guidelines on classification and offering specific examples on how the classification scheme should be applied. Severe experiments include those that have a serious impact on animals for any duration or a moderate impact over a long time. Severely impacted animals may experience devastating disease or even death as a potential experimental outcome.^33^ The United States Department of Agriculture (USDA) has defined pain/distress categories based on whether or not the animal experiences pain or distress; if pain or distress can be relieved with pharmacological interventions; or, the most severe category, experiments where the animal experiences pain/distress but interventions are withheld due to scientific necessity.^48^ The USDA Pain and Distress Categories document also includes a guide on how to classify specific experiments.^48^

Harm to animals is generally predictable as long as the experimental plan contains all necessary details of the procedures used in the animal studies.^49^ Based on knowledge of how these procedures may impact animals, prospective assessment of harm is possible and actions to reduce or eliminate harm (i.e. refinements) can be implemented. However, unpredictable experimental outcomes sometimes occur, and a retrospective review of an experiment can reveal useful information to aid planning in subsequent experiments of a similar type to avoid or reduce harmful circumstances.

Severity classification of animal experiments is helpful to the responsible entities when planning their review processes. As noted previously, mild procedures that are repeated frequently may also interfere with the animal’s ability to perform normal behavior or recover between procedures. Frequency of procedures is therefore relevant. An overview of procedures along a timeline can be schematized using an activity chart/map that records all the procedures for an animal along that timeline.^50^

The new reporting system for animal experiments in Europe includes a classification of experiments.^33^ Illustrative examples for the process of severity classification, day-to-day assessment and actual severity assessment are also provided.^47^ Declassification of experiments to lower classes is a way of monitoring performance in the refinement of experiments. Refinement is an otherwise complex concept involving technical insights and impact measures that are not as readily communicated to lay audiences as the simple reporting of numbers of animals used (reduction measures).^36^

Classification or quantification of the harm and benefit metrics of an experiment is a possible way of communicating with the public about the use of animals in research. The public is commonly focused on animal studies perceived as involving severe harm; however the majority of experiments are likely to be classified in the moderate or mild harm category. By successful implementation of refinement strategies severe experiments can be avoided.

## Benefits

An overview of benefit dimensions identified in the literature reviewed is presented in Table 3. Benefits identified in this review can be divided into three main dimensions: scientific quality, promise or potential outcome, and actual outcome.

**Table 3.** Benefit factors identified in literature review.

	Quality dimension				Promise dimension							Actual benefit dimension			
Reference	`Good science'}\cr \rot \hbox{dissemination of results	Originality	Aim and objectives	Realistic potential,}\cr \rot \hbox{feasibility	Benefits for humans	Benefits for animals	Benefits for}\cr \rot \hbox{environment	Economic interests	Health interests	`Surrogate outcomes' versus}\cr \rot \hbox{`health outcomes'	Secondary (long-term)}\cr \rot \hbox{benefits	Primary (short-term}\cr \rot \hbox{or direct)	Safety interests	Knowledge interests	Educational interests
Bateson 1986^1,6^	+														
Bateson 1992^3,7^	+				+	+	+	+						+	
Porter 1992^32^			+	+											
Mellor 1994^30^											+	+			
Stafleu 1999^40^			+	+				+	+	+				+	
Mellor 2004^31^											+	+			
Rickard 2004^5,6^	+				+	+									
Voipio 2004^7^	+			+	+	+	+	+	+				+	+	+
Schuppli 2004^3,8^	+														
FELASA 2005^41^	+	+	+	+											
Degrazia 2007^4,9^					+										
EU Directive 2010/63^2^	+		+		+	+	+							+	
Boddy 2011^2,6^	+										+	+			
Boyd 2012^2,5^										+					

Science and society, through regulations, have accepted the use of animals as surrogates for humans and as basic research subjects in diverse scientific endeavors regarded as beneficial to society, especially to improve human health, veterinary medicine, safety testing and to advance scientific knowledge.^8^ The use of animals for educational purposes has also been proposed,^7^ and a discussion of whether economic interest justifies the use of animals has also been raised.^7,37,40^

In Europe, potential benefits have to be described in a project summary intended for the public according to the consensus document providing guidance for the drafting and publication of non-technical project summaries.^2^ A template and an illustrative example have been published.^51^ Similar provisions can be obtained in the USA through the checklist of the protocol review delineated in the *NRC Guide*^1^ and through Principle II of the *US Government Principles for the Utilization and Care of Vertebrate Animals Used in Testing, Research and* Training.^21^ Researchers need to explain the potential benefits derived from investigations in research animals.

Benefits which can be categorized as ‘future promise’ may never be realized and it can be difficult to describe them precisely. ‘What, who, how and when’ have been suggested as key questions to ask to better address what the anticipated benefits are.^52^

Historically animals have been used as models for humans in studies of basic biological/disease mechanisms, product safety evaluations and development of new therapies. They have also been used frequently for studies in environmental/ecological science, agriculture/production enhancement and as models for disease studies in non-human species. These are all examples of benefits that accrue advantages to individuals, groups of individuals or societies at large that require animal use in scientific endeavors. Primary benefits of this nature would include the intrinsic value of knowledge itself and the relevance of this knowledge in applications that are directly beneficial to humans and other species or the global environment and that sustain the quality and diversity of life. Examples of primary benefits also include the impact of potential improvements in cancer therapy for a patient, with cancer targeted by that therapy. There are also examples where fundamental studies in a species can be extended to taxonomically similar invertebrates. The protection of cephalopods in Europe^2^ is such an example and is based on studies on that species and recommendations from scientists.^53^ In this case, the benefits apply to the class ‘Cephalopods’ (phylum Mollusca) as a group, rather than to the individual research animal. Some experiments might have benefits applicable to several domains. Experiments directed to improving efficiency in agriculture can have economic interests as the primary aim or benefit. According to Mellor^31^ economic benefits alone should not be used to justify animal experiments, especially when they cause much harm. However economic benefits for the farmer by reducing production costs can go alongside benefits for the environment by reducing pollution or by more efficient utilization of commodities, and also with improved health and well-being for the species in question. In some cases such studies can be a benefit for the actual animals involved in the study by providing them with improved conditions to maintain good health and well-being as well, and knowledge gained from these studies may benefit the farmers and stakeholders involved in the farming industry.

The secondary benefits identified are not necessarily dependent upon the data derived from sentient animal models and these benefits should not qualify as sufficient reasons for research in animal models causing harm in the absence of a compelling potential scientific reason. Economic benefit is an example of a secondary benefit. This does not only refer to the economic advantages accrued to a single researcher, but also to the economic benefits to the community built around the industry that commercializes or utilizes the scientific finding(s). For example in the cancer therapy scenario above, significant secondary benefits are the consequences for the health-care system of cost reductions (compared with cost overruns) resulting from new therapies, and/or families’ and society’s benefits from health improvements in family earners or the workforce generally. Also, the commercial potential derived from findings in animal research studies can result in products that will be available to improve the lives for many people or for animals, thus further increasing the benefit. The term ‘wider benefit’ has also been used for such secondary benefits.^26^ The potential for a high value research outcome (research quality factor) and the likelihood of research success (probability of achievement factor) have been included as valuation domains in the assessment of benefits. The likelihood of benefit will depend on the likelihood of the success of a research project in its methodological approach, data acquired, and data impact or applications.

Other examples of secondary benefits include improved organizational reputation and success related to productive scientific endeavors or enhanced career prospects, earning power, and consequently family benefits for individual scientists and their support staff needs. Secondary benefits can also include enhanced benefits for the wider community (the city, region or country) through economic, educational, social and other contributions made by the organizations, scientists and support staff who are part of the community. Increased recognition of the intellectual arena, the exercise of our creative imagination, rationality and problem-solving skills in the pursuit of the animal-based branch of science, which is part of our culture, are also examples of secondary benefits.

Humans involved as subjects in scientific research are cognizant of the risks. They offer informed consent to proceed with the knowledge that they, others with the condition under study or others of their species will have the prospect of benefiting from the outcome of the research. However, there are some exceptions where subjects are unable to give informed consent such as research in pediatrics, geriatrics, dementia, etc. In all these cases the risk is evaluated by a proxy (parent, guardian or committee) who makes a decision on their behalf. Animals cannot offer informed consent comparable with a competent person (as distinct from positive reinforcement training to accept minor procedures). Therefore the responsible entities must ensure that animal interests are accounted for. Animals enrolled in clinical therapeutic trials for naturally occurring diseases may experience a direct benefit from participating as study subjects. This may happen more often as a result of our improved diagnostic ability for selecting veterinary patients with relevant clinical disorders for use in proof of principle studies with our increasing ability to identify promising molecular targets and evaluate the safety of interventions in non-animal systems. Recent examples of veterinary patients benefiting in this fashion include canine cancer therapy evolving through molecular markers and pathway studies in human tumors and rodent models and regenerative stem cell therapies in companion animals developed through studies in rodents and other model systems.^54,55^

Undoubtedly, the rapidly accumulating progress in our understanding of the genetic and molecular basis of disease and therapeutic response through animal and non-animal model systems will allow more animals to benefit directly through their participation as research subjects in the future as we improve our ability to recruit appropriate target populations for study. However, in most instances animals have been used historically and will continue to be used in research studies or educational and testing applications as proxies for another species and while receiving no immediate direct benefit and with an unknown prospect of receiving a future benefit as part of the species.

As noted above, in some cases animals participate in research projects analogously to humans, without informed consent, that elucidate whether or not a new therapy will benefit them directly as active clinical patients. This type of research animal utilization may become more prevalent as molecular medicine and personalized medicine continue to advance. However, to limit scientific inquiry in animals only to these types of studies would be anathema to the interests of the global scientific community and society and inconsistent with the use of animals for other societal objectives.

Factors related to quality and potential, design of experiments and publishing have also been defined as benefit factors by some authors,^2,7,16,26,37,38,41,56^ and poor performances on these can lead to unreliable results or lack of dissemination of results and thereby reduces likelihood of any benefits. Without a plan for dissemination of results, realization or impact of a study is less likely.^26^

Based on the literature we have reviewed it seems that benefits, in contrast to harms, are less well defined with regard to classifications or means to strengthen benefits in operational terms. This can be explained by the fact that performing HBA in a systematic way and thereby defining and describing benefits is not common practice. For harm the 3Rs have been an operational algorithm for reducing harm since 1959.^5^ The quality of scientific experiments is definitely a factor that impacts benefit in the way that a well-designed experiment is a fundamental criterion for reliable information and for generating any benefit at all. Actual outcome benefits, like acquiring skills through training or safety testing in animals, have direct application; however in many situations alternative methods are available and the use of animals is therefore not justified. Independent of whether promised or potential benefits are realized – most agree that experiments contribute to increased knowledge and the understanding of a phenomenon. The question is then whether this knowledge is of such importance that animal use is justified.

## Balancing and comparing harms and benefits

In the previous sections we have defined and described the current concepts and elements of harm and benefit. We have summarized different dimensions of harm and benefit discussed in the literature reviewed in Tables 2 and 3. An HBA also includes a systematic way to compare and weigh the harms and benefits. In the literature reviewed there is a mixture of systems to categorize these parameters in lists of attributes or questions to be addressed in the analysis of harm and benefit. A summary of the models and their strengths and weaknesses are presented in Table 4.

**Table 4.** Summary of the strengths and weaknesses of different models of harm–benefit analysis (HBA).

	Strengths	Weaknesses
Categories	Categories are useful for simplifying a complex picture. Identify severe categories and stimulate actions to avoid them.	The categories do not fit all cases.
Algorithms	Algorithms are helpful in guiding a decision.	Moral dilemmas cannot/shall not be solved by arithmetics.
Graphic representations	Graphic representations have pedagogic value in visualizing the concept and relationship between harm and benefit.	Depend on defined categories. Not operational.
Process-oriented models	Process-oriented models structure the HBA process, how to balance different opinions and question quality of the analysis. Generic.	Do not provide an answer on what models to use or provide solutions for conclusions.

### Algorithm models

An algorithm is a process or set of rules to be followed in calculations or other problem-solving operations. Ideally, an algorithm should work in all situations, and providing the ‘correct’ solution in every case.

Stafleu et al. have presented a complex set of formulas for calculating scores for harm and benefit.^40^ This study has distinguished itself from other studies by providing a more complete model for scoring benefits in a systematic way as it has provided a set of formulas.^40^ Other mathematical models describing harm–benefit as fractions or as a sum have also been described to illustrate the HBA concept on balancing harms and benefits.^57^ However these models did not provide an algorithm on how to estimate the size of harms and benefits. Since there is no common ‘currency’ or summary metric that reflects harm and benefit in a way that makes it possible to compare these sizes,^37,58^ others prefer a non-numeric scale using letters to avoid the apparent and misleading precision of arithmetic assessment.^31^ Other authors are not in favor of mathematical models because they give a false impression of objective accuracy.^7^

Mellor^31^ has stressed that harm–benefit evaluations cannot be reduced to an arithmetic exercise. In the Mellor et al.^30,31^ model, harms are categorized using letters (O, A, B, C and X, where X is the most severe category) instead of numbers. This was done intentionally to avoid any temptation to use arithmetic to draw a conclusion.^30,31^ Evaluation in the Mellor’s model is made based on the impact of the experiment on each dimension of the five freedoms.^29^ The greatest anticipated compromise specific to each domain is used as a final grade. Mellor^30,31^ discriminates between primary (or direct) benefits and secondary (or indirect) benefits. Grade A can be justified by both primary and secondary benefits, while B, C and X can only be justified by primary, direct benefits.^31^

### Graphic representations

Graphic models help the reviewer visualize the relation between harm and benefit using a graphical illustration such as a figure or a diagram. The first published report of a practical approach to conducting a general HBA in animal research studies can be credited to Bateson in 1986^1,6^ and based upon his work the role of the HBA first appeared in national guidance documents in the UK Animal Scientific Procedures Act in 1986. The UK subsequently incorporated the requirement for an HBA in the ethical review process and several other countries including Norway, Brazil, Tanzania and Australia have since adopted similar provisions.^59^

The Bateson square is an example where harm and benefit are presented along the two axes.^16^ Common traffic light colors have been added to indicate the favorable/unfavorable status of a dimension: green means acceptable; yellow signifies attention; and red indicates stop. Bateson also introduced research quality as a factor in the third dimension (*z*-axis) and thereby introduced a 3D cube model.^16^ More complex figures like flow charts and decision trees can be used to include more dimensions.^57^ However, as soon as the model becomes too complicated it loses its power as a simple graphic presentation.

The expert working group for the European Comm-ission on project evaluation and retrospective assessment has developed a modified Bateson cube.^52^ Bout et al. have presented a refined model based on Bateson that has been used for HBA in The Netherlands.^9^

Models like the Bateson model have been widely recognized for their pedagogic value to illustrate the concept of HBA. They are normative, illustrating that high harm and low benefit projects should be rejected. However, they are not necessarily operational so they cannot always be applied productively in challenging situations commonly encountered by oversight bodies. To be able to do this, there must be a clear definition of the different categories and scales, so that input information is represented properly in the graphic model.

In addition to Bateson, several other authors have addressed the process of HBA in animal studies, providing the responsible entity with a broad range of helpful suggestions to consider in constructing their own systematic approach to HBA.

### Checklists and key control questions

Some authors have also delineated useful checklists of keywords^7^ or key questions^57^ categorized under harms or benefits that responsible entities might find useful for inclusion in the development of an HBA. Authors generally have elaborated more expansive lists of harms than of benefits, and most authors have agreed that pain, injury or disease^2,7,30–32,37,38,40,41,49,60^ and fear, anxiety or distress^2,7,16,26,30,31,37,38,40,41,49,57,60^ are important harm factors (Table 2). The numbers of animals used, adverse alteration of the animals’ environment and husbandry, impediments to normal behavior, duration of study, species and prospect of death are also frequently cited harm factors in the literature review (Table 3).

### Process-oriented models

**Figure 1.**
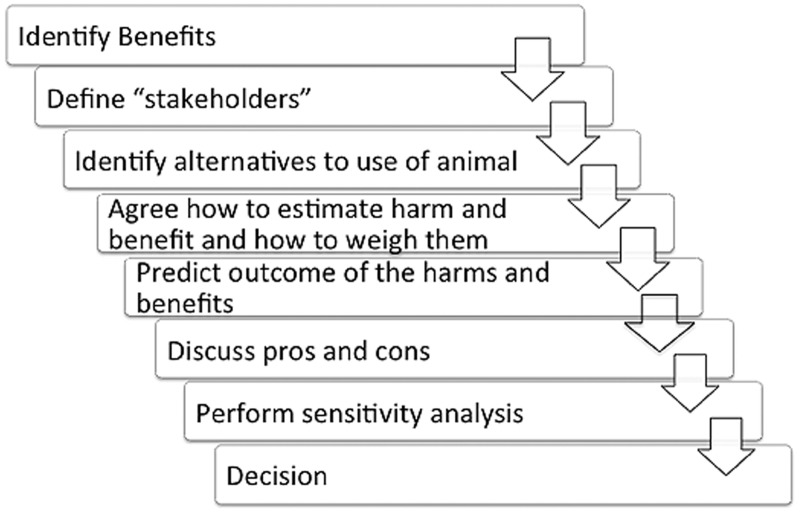
A generic harm–benefit analysis.

While the models above focus on different ways to categorize and present HBA, a process-oriented model, or sequential-question model^61^ focuses on the process of how information is achieved and evaluated. This also includes the competence of persons involved in the process. In Figure 1 we describe a list of steps as an example that comprises a generic HBA.

Process-oriented models are generic and are structured around the HBA process. They safeguard how different opinions are represented and question the method and quality of the analysis. Process-oriented models do not provide answers on what model to use or provide solutions for conclusions.

## Discussion

Classifying experiments in severity categories aids the identification of harmful experiments that require extra attention or resources. Such classification systems have been developed both in Europe^46^ and in the USA.^48^ Experiments causing severe harm warrant careful evaluation and the incorporation of applicable refinements such as the selection of alternative experimental parameters of satisfactory value, early intervention and other more humane endpoints. Such refinements can also be used as means to down-classify a specific experiment from a severe category to a moderate or mild category, with the overall goal of maximizing harm reduction in all cases as the objective in mind. Categorization also helps in the communication on how HBAs and evaluations are made in a transparent manner. In general most models depend on clearly defined categories for harm, referring to a severity scale that enables a comparison of harms. Even if severity categories are well-defined, complexities remain in the estimation of harm levels.^60^

Responsible entities conducting HBAs should expect to encounter dilemmas on a regular basis. For example, the prospective analysis of many studies will involve the estimation of harm based upon similar cases or information from the literature or colleagues and such estimations may not predict outcomes sufficiently. Further, if a tendency towards utilization of a general or universal categorization system versus evaluating each particular case evolves over time, responsible entities conducting such reviews must remain attentive to the potential vagaries of each approved study/procedure to ensure prompt corrective actions if animal welfare is compromised. For example, a study involving a highly invasive procedure (surgery) categorized as severe and is performed by a highly proficient expert might be less harmful and fully permissible whereas a moderately severe procedure performed by someone less skilled may be perilous for the animal subject. Another example from the area of infectious disease would be the use of a standardized model of infection in which the dose of infectious agent, pathogenicity of the specific agent used, housing conditions and the associated treatments studied could have a profound influence on animal welfare. These distinctions deserve careful scrutiny in a thorough HBA. Also, they serve to re-emphasize that prospective HBAs should always be reconciled with an actual HBA performed retrospectively to help guide future decision-making by the responsible entities.

The species used has also been considered as a harm factor. Experiments using some animal species are regarded as more harmful than other species even within the same subphylum (vertebrates). There are some special restrictions for the use of non-human primates in Article 8 of the EU Directive.^2^ Russell and Burch also stressed the importance of using less sentient animals as an alternative.^5^ Extensive research studies have illustrated that phylogenetically lower animals have advanced social systems, communication and collaborating systems. Some regard fishes as less sentient animals and zebrafish have been used as an alternative model for mammals in studies of human disease.^62^ However welfare states associated with the use of fishes are an emerging issue in international science programs.^63^ Cephalopods are a group of animals that have recently been included and protected by the new EU Directive.^2^ Research on these animals has discovered that they can not only experience pain, but they can also have cognitive abilities similar to what are recognized in advanced vertebrates.^53^

Harm is relevant for sentient animals or animals that can experience pain. Whether or not modifying an animal’s genome is a harm factor depends on the ethical positions of those involved in the debate. Animal welfare advocates will only regard this as harmful if the modification results in a deleterious phenotype. A survey from Denmark showed that severe discomfort was only the case for about 15% of all genetically-modified animal strains, while 64% showed no discomfort.^64^ The ability to suffer is a relevant harm factor. However, ‘ability for suffering’ requires consciousness in order to experience the harm. This has the implication that harm is only relevant for autonomously living animals and not a cell or an embryo.^39^ Progress in gene technology has created a new ethical dilemma by facilitating the incorporation of new xenogeneic traits resulting in potentially deleterious phenotypes unknown in the natural history of the genetically-manipulated species. The level of consciousness of the genetically-manipulated species and the harm impact of the induced phenotype may warrant consideration in these cases. It is important to recognize that there might be conflict between a researcher’s perception and the public’s perception of what is ethically relevant.^39^

As harm is a result of planned activities, it can generally be estimated in advance, hence providing an intrinsic opportunity for the implementation of harm control measures (i.e. refinements). By contrast, benefit is more poorly defined; benefits have been categorized in domains, but not ranked in value comparable with the severity categories for harm. There is no clear hierarchy for benefits, with the exception that economic benefits or the benefits associated with improving vanity products would warrant less support than health benefits for severe diseases such as cancer or cardiovascular disease in humans.^10,65^ Surveys indicate that the benefits of progress on less severe diseases or lifestyle diseases – such as obesity – are also less supported.^10^ However, public opinion is often based on a simplified picture of the causality between a disease factor and disease, not reflecting the actual complexity behind a certain condition such as obesity, for example.^65^ In such cases, researchers will be challenged and will have to explain very clearly what they want to achieve by using animals and why alternative approaches cannot provide equally valid information, while such a justification will be easier to sell in the question of a study of cancer, for example. Even if weighing benefits for different purposes against each other is difficult, Stafleu et al. have made an attempt to include benefit weighing in their model for HBA.^40^

Benefits of a particular project depend upon internal factors such as scientific and technical quality. Actual benefit also depends on external factors such as the usefulness of the data for immediate application or commercialization, or to future productive scientific endeavors ultimately deemed valuable by society. Although there are frequent instances where the researcher believes that a high value benefit is within reach, it is much more common for the beneficial value of the anticipated experimental outcome to be unknown. While harm is immediate, the certainty of a benefit can be unpredictable and intangible.^26,49^

In the EU Directive, potentially beneficial outcomes in research, education, testing and disease diagnosis may justify granting permission to use animals but only when there are no alternatives to animal use.^2^ There is inherent uncertainty regarding the direct outcome and applicability of basic research. The investigation of basic mechanisms that are unknown or only partially characterized cannot be reliably conducted using alternative methods. While basic research is burdened with some uncertainty regarding direct benefits, we have a long history of experience showing that basic research is beneficial for the development of society, especially with regard to taking advantage of technological progress. For routine tests and educational activities, many alternatives are available and more appear to be evolving. Also, guidelines for the use of animals in educational applications have long contained the proscription that harm should not be inflicted solely for the purpose of educational or instructional activities. Learning outcomes and improvements in skill can be direct benefits of practicing procedures in a living animal. However, using animals for training purposes can be more easily replaced by alternatives than is the case with basic research.

There seems to be a stronger tradition of emphasizing harm, especially of harm reduction, than of considering benefits in responsible entities.^66,67^ In a survey among workshop participants at a FELASA meeting (2010, Helsinki, Finland, unpublished; Table 5) 90% agreed or strongly agreed that 3R implementation is a fundamental ethical issue; however only 16% thought that the 3Rs are equally balanced. Most (81%) agreed that refinement is the R that got the most attention, and 90% agreed or strongly agreed that harm to the animals is a fundamental issue in ethical evaluations. Also the majority (71%) agreed that the benefit for humans is a fundamental issue in ethical evaluation and that harm versus benefit (75%) is important in ethical evaluations. Only 38% reported that the right for humans to use animals in research is a major part of the discussion in ethical evaluations. In this audience, which presumably was more insightful and pragmatic than the general public on the matter of animal use in research, only 15% agreed that philosophical issues are an important part of ethical evaluations. Other studies have reported that discussions nearly unilaterally focus on details and technicalities more than on overall ethical judgments in the approval of animal research.^9^

**Table 5.** Topics and discussion in ethical review of animal experiments.

Agreement with the following statements	Very much disagree or disagree	Neutral	Very much agree or agree
3R implementation is a fundamental ethical issue.	6%	5%	89%
Ethical committees, in my experience address all 3Rs in an equally balanced way.	53%	31%	16%
Ethical committees, in my experience, use most time discussing Replacement.	57%	33%	10%
Ethical committees, in my experience, use most time discussing Reduction.	21%	46%	33%
Ethical committees, in my experience, use most time discussing Refinement.	19%	0%	81%
The harm to the animals is a fundamental issue in ethical evaluation.	10%	0%	90%
The benefit for humans is a fundamental issue in ethical evaluation.	14%	14%	72%
The harm versus benefit is a fundamental issue in ethical evaluation.	10%	15%	75%
Scientific quality is an important part of ethical evaluation.	20%	15%	65%
Scientific issues are a major part of discussion in ethical evaluation.	29%	19%	52%
Philosophical issues are an important part of ethical evaluation.	60%	25%	15%
Technical issues are an important part of ethical evaluation.	11%	28%	61%
The right for humans to use animals in research, is a major part of discussion in ethical evaluation.	52%	10%	38%

Olson A, Kalman R and Brønstad A. Survey from FELASA workshop on ethical evaluation, Helsinki, Finland, June 2010.

The focus on harm in animal ethical review bodies might be because harm seems easier to assess for the reasons mentioned above. However, it might also be explained by the composition of the typical animal ethical review bodies having more competence and ease in the consideration of technical and harm issues. Evaluating benefits is more commonly performed by funding bodies which operate in a separate domain disconnected from the responsible entities. The split evaluation of harm versus benefit between these two separate authorities may alter the value and outcome of the HBA.^66,67^

The consideration of benefits in the HBA of animal studies shares some features with the consideration of benefits in the HBA of human clinical trials. However, in animal experiments, benefits seem most difficult to assess, while in human clinical studies harms are regarded as the more difficult part.^25^ This might be because some uncertainty of risk for human participants has been eliminated in preclinical testing and the prospect of residual harm is scrutinized in detail, and because there is a chance that the participant will benefit from a new drug or treatment regimen.

Professional terminology for the discussion of harm is well established, i.e. 3R, severity classes, refinements, harm reduction, humane endpoints are all expressions used in the discussion of harm especially regarding harm minimization. A similar terminology for the discussion of benefits seems to be lacking; and benefits are discussed in more general terms, i.e. benefits for certain groups or purposes, but not in terms of actions to increase benefits. In our opinion, it would be in the interest of stakeholders who have a need to use animals to develop a wording to highlight the anticipated benefits for their animal experimentation.

Several models for categorizing harm–benefit and algorithms on how to balance these have been presented. We categorized the different models as algorithm models, graphic models (squares, cubes and decision trees), checklists and key questions and process-oriented models. All these models depend on some kind of categorization to simplify a complex picture into defined units that can be used as input information in the model of choice. Simplifications in categories and models to aid the HBA may detrimentally reduce: the quality of information, appreciation of the uniqueness of each proposal analyzed, moral sensitivity and attention of the responsible entities. Simplification may also favor routinization on the cost of cultivating an ethical vocabulary and creating distance to consequences of actions.^68^

HBA is dependent on the context, and context changes over time. Seventy-five years ago animals were used for pregnancy testing, such as the Friedman test in rabbits developed in 1939.^69^ It was beneficial for women to know if they were pregnant or not. Today, in vitro kits for pregnancy testing are cheap and easily available commercially and women can carry out the test themselves. So even if it is still a benefit for women to know if they are pregnant or not, the use of animals for pregnancy testing is no longer morally acceptable because alternative methods have been developed. Decades ago central venous catheters were surgically placed routinely without imaging the final position of the catheter tip, causing complications from endocardial damage. Under what circumstances would conducting this procedure in this manner be considered acceptable today? Harm–benefit evaluations are dependent on the context and availability of alternative methods. Such alternative methods should be checked out at an early stage before large efforts are used in harm–benefit evaluations. Decisions in the past cannot take precedence over decisions in the future because technological solutions as well as research questions that need to be answered will change as research and technological developments advance. For ethical evaluation of an animal experiment, the context in which the animal experiment is done will always be relevant.

Some AEC members expressed that they have always been doing harm–benefit evaluations while others expressed concerns that doing HBA is something new and that it changes the role of the AECs.^67^ Though models to illustrate the harm–benefit concept like the Bateson cube^16,37,52^ are known, such models are not operational as long as they lack clear explanations on what to put in the different boxes in the model. Algorithm models^32,40^ are more instructive but are criticized for reducing moral questions to arithmetics.

Although harms and benefits have been systemized and categorized, and there may be common understanding and agreement on these categories, weighing harms and benefits will be biased depending on the individual persons responsible for the discussion.^27^ Attitudes to animal experiments influence decisions of being in favor or disfavor of particular animal experiments.^10^ Savadori et al. have demonstrated that such biases, also called affective heuristics, do not only influence the decisions of lay people.^14^ Affective heuristics also influence opinions of experts, however to a lesser degree than to lay people.^14^ Based on this fact it might be reasonable to suggest that different interests and/or competencies should be present in the discussion of HBA to enlighten different perspectives and views of a particular case. A broad representation of interest minimizes some – but never all – biases caused by affective heuristics.

A reason why people find harm–benefit evaluation difficult is that it actually challenges personal attitudes to animal experiments, to animals as sentient beings and to the relationship between animals and humans. Anyone who is part of such decision-making is responsible for this, and this is different from ticking checkboxes, checking compliance with regulations, guidelines or the 3R principles.^66,67^ Compliance with regulations is required but does not encompass the totality of ethical behavior. Ethical responsibility is a domain placed higher in the hierarchy than legal responsibility.^70^ Sometimes regulations have limitations in providing a good solution and there is a need for making a ‘wise’ decision for a particular case. This is an important decision for the responsible entities.

**The working group defines HBA as a transparent systematic method to gain information about harm to animals and expected benefit so that qualified decision of approval or rejection of projects can be made**.

Ethical ‘tools’ are different ‘rules’ used to justify or solve moral dilemmas. An example of such a tool is the 3Rs.^5^ These are intended to minimize harm to animals where possible by using alternative biological systems or replacement methods; to reduce the numbers of animals used through animal model selection and in vivo methods that improve data compactness; or procedural refinements to reduce suffering. No ‘tool’ or model is perfect, and though the 3Rs have gained support by moving animal experiments in a better direction there are still several conflicts hidden within the 3R principles.^36^ Furthermore, unilateral focus on the 3Rs draws attention towards harms and the negative aspects of animal experiments, while not taking the potential positive outcome (benefits) or context into consideration.

**Box 5.** Ethical stances explaining the success of HBA.

Peter Sandøe (personal communication)
1. A utilitarian perspective where equal needs should be treated equally, irrespective of whether these needs are human or animal – so harms to animals are only allowed if they are justified by a higher likely benefit (and if there is no alternative line of action with a better harm–benefit balance).
2. A contractarian perspective where HBA is all about ensuring minimizing animal harm and maximizing human benefit, without questioning the basic speciesist premise that animals are for us to use, and without really trying to compare the size of the animal harm to the likely human benefit.

HBA serves as a foundation or comprehensive ethical maxim, which subsumes later application of ethical tools such as the 3Rs in the construction of humane animal experiments. To justify studies that are anticipated to inflict harm to animals, there must be a reasonable expectation of specific benefits commensurate with the level of harm; or there must be an urgent scientific need with profound implications, despite the suffering caused, that cannot be fulfilled using other methods.

However, harm and benefits are not necessarily the only considerations made in the final decision. Kvalnes and Øverenget proposed the‘navigation wheel’ as a model for decision-making to keep track of relevant decision-making factors that also include factors other than ethics in decision-making.^71^

The utilitarian concept of justifying the use of animals in research has existed for decades and also seems to be applied independently of attitudes to animal experiments.^10^ Utilitarianism or consequentialism is an ethical philosophy holding that the proper course of an action is the one that maximizes utility (i.e. maximizing happiness and/or reducing suffering).^8,72,73^ Jeremy Bentham and John Stuart Mill were important contributors to classic utilitarianism. Singer, an Australian moral philosopher specialist in applied ethics and author of the book *Animal Liberation*,^34^ also holds a preference for the utilitarian perspective. According to Singer the interests of animals should be considered because of their ability to suffer, and he argues that animals should have rights based more on their ability to feel pain than on their intelligence. Ability to suffer was also the main relevant property according to Jeremy Bentham (1748–1832):

‘*The question is not, Can they reason? nor Can they talk? But can they suffer?*’,

Ethicists may disagree that HBA is uniquely utilitarian as utilitarian ethics is often based on the assumption that the welfare of animals and humans is equally important. However, when performing an HBA in the context of animal experiments, human health and welfare interests usually count for more than animal health and welfare interests. Though HBA is based on maximizing utility, it also has elements of contractarianism, an ethical position placing human or one’s own interests first.^74^

One of the success factors of HBA is that it makes sense from different ethical perspective. However this can also be a source of conflict as different people, with different stances, are influenced by affective heuristics^27^ and therefore have different expectation as to what HBA can offer, and this thereby gives rise to new controversies rather than solutions.

An important side-effect of performing a thorough HBA is that the responsible entity and the scientist work carefully to incorporate the 3Rs to the fullest degree compatible with the scientific question being posed. This collaborative commitment to the 3Rs likely helps foster a positive public perception of animal use in research.

Regulatory compliance provides a license to operate. Regulations are commonly based on past knowledge, and may therefore be out of date. While regulatory compliance is demanded, and is important, it may only contribute minimally to the formulation of broader ethical decisions. We expect that doing harm–benefit assessment will stimulate scientists to investigate alternative experimental approaches in light of the current context. In our opinion HBA stimulates ethical reflection, dialogue and discussion on the use of animals in research. Assessment of harms and benefits enables researchers, reviewers and funders to decide whether a particular experiment is worth doing at all.^26^

## Conclusion

The AALAS–FELASA WG on HBA has reviewed the existing literature on this topic and defined the current concept and elements of HBAs. Harm is a negative impact on the sentient being. There is consensus that sentience and ability to suffer are relevant. There is also agreement that harm is more than pain and suffering, and includes all sources that can cause negative impact on animals, and harms can be related to all domains provided by the five freedoms. Disrespect for life is a harm factor. Some authors also discuss genetic manipulation as an infringement in itself, while others regard this as harmful only as long as the genetic modification causes an impaired phenotype. Designing experiments performed with total anesthesia where the animal will not regain consciousness is regarded as a refinement and a way to limit harm experiences.

Benefits for human, animal or environment health are regarded as acceptable benefits to justify animal use as long as there are no alternatives. The intrinsic uncertainty whether promised benefits will be realized or not, must be compensated by strengthening the quality of the study to optimize the possibility of reliable known benefits.

The quest for knowledge, safety and forensic purposes are also regarded as legitimate justification. Economic interests or benefits related to vanity products are less acceptable benefits; however such studies may also have other favorable secondary benefits. Primary or actual benefits seem easier to accept than secondary benefits because the relation between the experiment and outcome seems more likely.

Several models for comparing and weighing harm–benefits are presented. The Bateson square and cube have gained recognition as models to illustrate the concept of HBA. This is a good model for illustrating the concept, but is less operational as long as there are no clear rules for what to put in different cells in the cube. Algorithm models have also been presented but are criticized for reducing ethical issues to arithmetic exercises. Checklists and key questions are useful for checking that important relevant harm and benefit factors are addressed. Subjective opinions will influence decisions in a committee responsible for HBA. A broad representation of different legitimate interests in the decision-making group is therefore recommended, as provided in a process-oriented model. Whatever model is chosen it should be transparent so that it is possible to verify what harm and benefits were evaluated and on how much weight is put on each of them. Finally, the best solution may be found by combining aspects from the different models.

**The working group defines HBA as a systematic, transparent way to assess and compare harms, benefits and how they are balanced**.

HBA is valuable in that it stimulates ethical discussion and reflection; it questions current practices and is therefore a driving force for improvement and ethical decisions. HBA identifies harm and drives researchers to seek alternative approaches to reduce or eliminate harm (refinement), and is important in avoiding uncritical use of animals just for the cause of the good. HBA is based on the assumptions of maximizing utility for the majority where human interests count most and is an essential part of the ethical review. Since HBA drives ethical reflection and discussion on current practices, it is important for building public support to ensure that harm to animals is taken into consideration and that animals are only used to achieve legitimate important benefits. Decisions based on a particular HBA are dependent on and limited to the current context.

In Part 2 of the WG report a method for analyzing harm/benefit is provided.
